# Value of the short physical performance battery (SPPB) in predicting fall and fall-induced injury among old Chinese adults

**DOI:** 10.1186/s12877-023-04290-6

**Published:** 2023-09-18

**Authors:** Weiqiang Li, Zhenzhen Rao, Yanhong Fu, David C. Schwebel, Li Li, Peishan Ning, Jiaqi Huang, Guoqing Hu

**Affiliations:** 1 Department of Epidemiology and Health Statistics, Hunan Provincial Key Laboratory of Clinical Epidemiology, Xiangya School of Public Health, National Clinical Research Center for Geriatric Disorders, Xiangya Hospital, Central South University, Changsha, 410078 China; 2 Department of Psychology, University of Alabama at Birmingham, Alabama, 35294 United States of America; 3 National Clinical Research Center for Metabolic Diseases, Metabolic Syndrome Research Center, Key Laboratory of Diabetes Immunology, Department of Metabolism and Endocrinology, Ministry of Education, The Second Xiangya Hospital of Central South University, Changsha, Hunan 410011 China; 4 National Clinical Research Center for Geriatric Disorders, Xiangya Hospital, Central South University, Changsha, China

**Keywords:** Short physical performance battery (SPPB), Falls, Fall-induced injury, Prediction, Community-dwelling, Older adults

## Abstract

**Background:**

The short physical performance battery (SPPB) is an easy-to-use tool for fall risk prediction, but its predictive value for falls and fall-induced injuries among community dwellers has not been examined through a large-sample longitudinal study.

**Methods:**

We analyzed five-round follow-up data (2, 3, 4, 5, 7 years) of the China Health and Retirement Longitudinal Study (CHARLS) (2011–2018). Data concerning falls and fall-induced injuries during multi-round follow-ups were collected through participant self-report. The Cochran-Armitage trend test examined trends in fall incidence rate across SPPB performance levels. Multivariable logistic regression and negative binomial regression models examined associations between SPPB performance and subsequent fall and fall-induced injury. The goodness-of-fit and area under the receiver operating curve (AUC) were used together to quantify the value of the SPPB in predicting fall and fall-induced injury among community-dwelling older adults.

**Results:**

The CHARLS study included 9279, 6153, 4142, 4148, and 3583 eligible adults aged 60 years and older in the five included follow-up time periods. SPPB performance was associated with fall and fall-induced injury in two and three of the five follow-up time periods, respectively (*P <* 0.05). The goodness-of-fit for all predictive models was poor, with both Cox-Snell R^2^ and Nagelkerke R^2^ under 0.10 and AUCs of 0.53–0.57 when using only SPPB as a predictor and with both Cox-Snell R^2^ and Nagelkerke R^2^ lower than 0.12 and AUCs of 0.61–0.67 when using SPPB, demographic variables, and self-reported health conditions as predictors together. Sex and age-specific analyses displayed highly similar results.

**Conclusions:**

Neither use of SPPB alone nor SPPB together with demographic variables and self-reported health conditions appears to offer good predictive performance for falls or fall-induced injuries among community-dwelling older Chinese adults.

**Supplementary Information:**

The online version contains supplementary material available at 10.1186/s12877-023-04290-6.

## Introduction

As the population ages, elderly falls have become an increasingly important public health challenge worldwide [1–3]. Predicting risk for falls is desirable to identify high-risk individuals and support early implementation of appropriate fall prevention strategies [4–6].

The Short Physical Performance Battery (SPPB) is a widely-used fall predication tool, and was recently recommended as a risk assessment tool for fall prevention and management of older adult health in the World Falls Guidelines, which was developed by a World Falls Task Force comprised of 96 multidisciplinary experts representing 39 countries and 36 scientific and academic societies [7].

Although two prospective studies report significant associations between the SPPB and the risk of falls and fall-induced injury among older patients in a hospital setting [8] and among older adult outpatients [9], evidence about the value of SPPB in predicting future falls and fall-induced injuries remains disputed among older adults living in the community. A cross-sectional study of 2710 community-dwelling adults conducted in Italy [10] and a 4-year follow-up study of 417 community adults conducted in the United States [11] both report a significant relationship between poor SPPB performance and a high fall risk. However, three other published prospective studies did not detect significant associations between the SPPB and the morbidity of falls and fall-induced injuries. One of those studies, in the United States, included 755 community-dwelling adults [12], and the other two, in Italy and Sweden respectively, had sample sizes of 567 and 202 [13, 14]. The performance of SPPB in predicting the risk of elderly falls and fall-induced injury has not been quantitatively assessed in published research among old community dwellers through a nationally representative and long-term longitudinal sample. Further, due to inadequate sample sizes, no published studies examine sex- and age-specific predictive performances of SPPB for subsequent falls or fall-induced injuries.

To address these knowledge gaps, we obtained data from the nationally representative China Health and Retirement Longitudinal Study (CHARLS) and assessed predictive performance of SPPB for both falls and fall-induced injuries among the full sample and among subsamples by sex and age group at five follow-up assessment points.

## Methods

### Data source

Data for this study were obtained from the China Health and Retirement Longitudinal Study (CHARLS), a nationally representative longitudinal study of Chinese residents aged ≥ 45 years. The CHARLS conducted baseline surveys in 450 urban and rural communities of 150 counties from 28 Chinese provinces [15] and then performed follow-up surveys every two or three years. The CHARLS collected information concerning demographics, family characteristics, individual health behavior, and health status, as well as retirement information [16]. Details of the CHARLS study are available at the study’s official website, http://charls.pku.edu.cn/en/.

We analyzed the publicly-available CHARLS data collected at three baseline time points (2011, 2013, 2015) and from five corresponding follow-up time periods: 2 years (2011 baseline to 2013 follow-up assessment; 2013 baseline to 2015 follow-up assessment), 3 years (2015 baseline to 2018 follow-up assessment), 4 years (2011 baseline to 2015 follow-up assessment), 5 years (2013 baseline to 2018 follow-up assessment), and 7 years (2011 baseline to 2018 follow-up assessment).

**Fig. 1:**
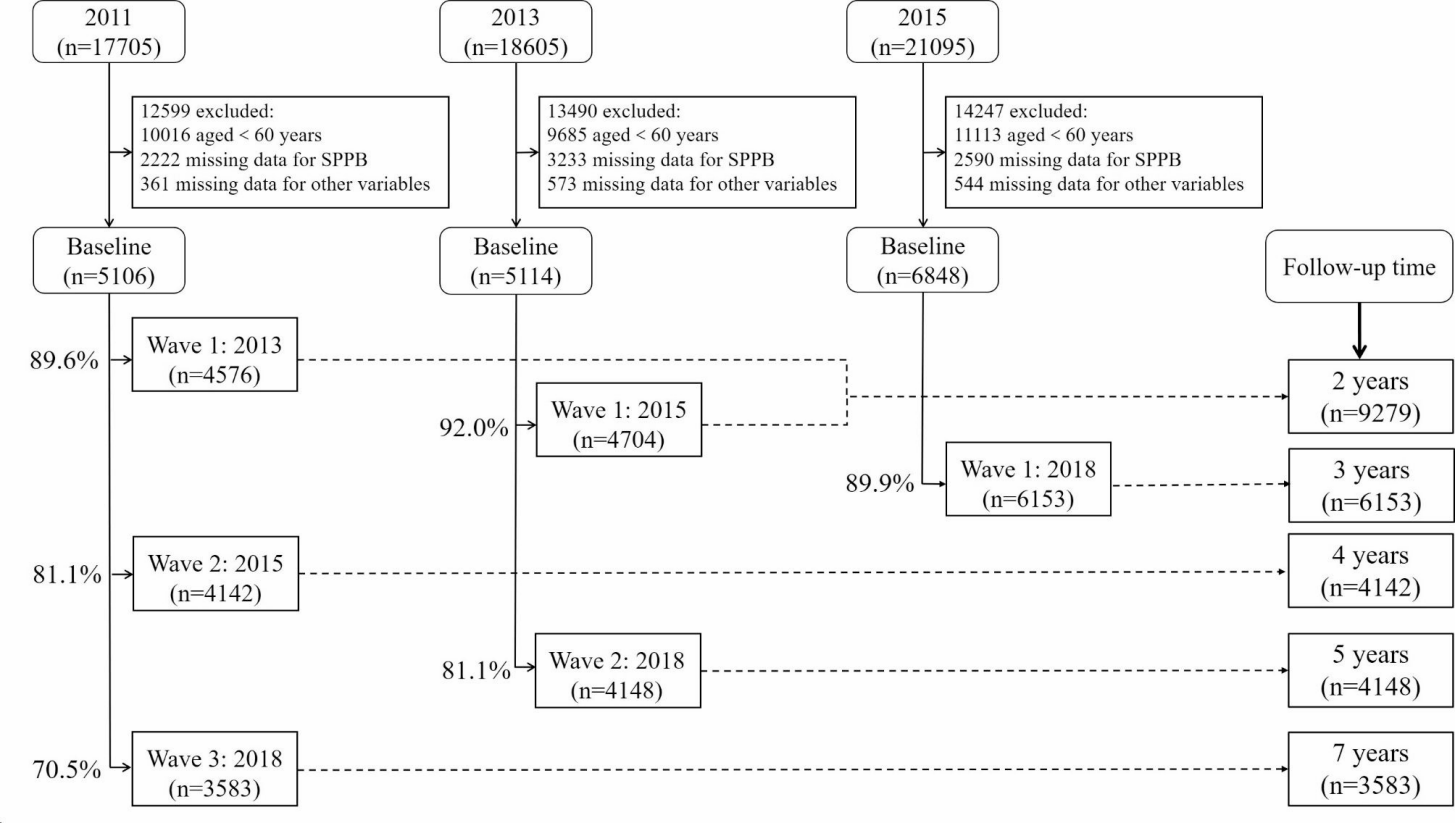
Assessment points, sample sizes, and inclusion details for the sample based on data publicly available in the China Health and Retirement Longitudinal Study (CHARLS)

Eligible participants for our study were limited to community dwellers aged ≥ 60 years when they joined the CHARLS study. We excluded participants from analysis for any of the following reasons: (1) age less than 60 years; (2) missing values for the SPPB or included covariates; and (3) no follow-up data available, either due to death or failure to complete surveys. Figure 1 describes the sample sizes at each assessment period as well as details of participant inclusion.

### Outcome measure

We considered two outcome events of interest, self-reported falls and fall-induced injuries that required medical treatment over the past two or three years. The CHARLS recorded falls as a binary variable (yes or no) based on a single self-reported question, “*Have you ever fallen down during the follow-up time period?*”. Fall-induced injuries were assessed as a discrete count variable (number of fall-induced injuries) through a single self-reported survey question, “*How many fall-induced injuries warranting a medical treatment did you experience during the follow-up time period?*”.

We calculated the incidence rate of our two outcomes as:

Fall incidence = (number of people falling / number of person-years) * 100%.

Fall-induced injury incidence = (number of fall-induced injuries / number of person-years) * 100%.

#### Short physical performance battery (SPPB)

The SPPB includes three tests: walking speed, repeated chair stands, and balance tests. Each test is scored from 0 to 4, with higher scores indicating better performance. Implementation details and the scoring system to administer the three tests in Chinese were described by Zhong et al. [17]. According the SPPB manual [18], the sum of the three test scores, which has a possible range of 0 to 12, is recommended to be categorized into three outcome groupings: low (score of 0 to 6), medium (score of 7 to 9), and high (score of 10 to 12). All CHARLS participants completed the SPPB test in Chinese under standardized administration by trained assessors at the time of their baseline assessment.

### Covariates

Based on the relevant literature [19, 20] and data availability in the CHARLS study, we considered the following variables as covariates:

Sex: male, female.

Age group: 60–69 years, 70 years and older.

Self-reported history of stroke or not: yes, no.

Self-reported memory-related disease: yes, no.

Activities of daily living (ADL) impaired or not: yes, no. ADL was assessed using the self-reported Basic Activities of Daily Living (BADL). The BADL includes 6 items and has good reliability and validity [21]. Participants were coded to be ADL-impaired if they reported difficulty or inability to perform any activity item [22].

Self-reported history of fall 2 years prior to the baseline survey: yes, no. This information was assessed via self-reported history of fall history in the 2 years prior to completing the baseline survey.

Depression: yes, no. Depression was assessed using the 10-item version of the Center for Epidemiological Scale (CESD-10) [23]. Participants were classified as having depressive symptoms (CESD-10 score ≥ 10) or not (CESD-10 score < 10) [24].

Sensory status: yes, no. Sensory status was assessed via self-report of hearing loss and vision loss and was divided into four groups for analysis: no sensory loss, hearing loss, vision loss, and both hearing and vision loss [25].

Muscle weakness: yes, no. Muscle weakness was evaluated by measuring the maximum force created by each hand (two trials for each hand using a dynamometer from a standing position). If scores were ≤ the 20th percentile of the weighted population distribution after adjusting for sex and body mass index (BMI) [26], based on prior reports from the CHARLS data [27], muscle weakness was coded as present.

Cognitive function: quartiles. Cognitive function was assessed using the American Health and Retirement Study (HRS) scale [28], which includes four dimensions: orientation, memory, computation, and drawing [29]. Participants were divided into four groups based on the quartile of their performance within the sample (0 = < *P*_*25*_, 1 = *P*_*25*_-*P*_*49*_, 2 = *P*_*50*_-*P*_*74*_, 3 = ≥ *P*_*75*_).

Detailed data concerning all variables appear in Appendix Table 1.

### Statistical analysis

The Cochran-Armitage trend test was used to examine the significance of incidence rate changes across the three SPPB performance groups of low, medium, and high; we expected an inverse relationship, with low scores associated with higher fall and fall-induced injury rates. We then fitted multivariable logistic regression and negative binomial regression models to examine the significance of SPPB performance in predicting the occurrence of falls and fall-induced injuries, respectively, after adjusting for sex, age group, ADL, history of fall in the past 2 years, depression, stroke, memory-related disease, sensory status, muscle weakness, and cognitive function. Adjusted odds ratio (OR), incidence rate ratio (IRR), and their 95% confidence interval (95% CI) were respectively calculated based on the multivariable models to quantify the associations of interest.

To quantify the predictive performance of SPPB, we separately fitted logistic regression models using SPPB performance as a single predictor and using SPPB performance and other risk factors together as predictors to compare the goodness-of-fit of different predictive models using Cox-Snell R^2^, Nagelkerke R^2^, and accuracy. Subgroup analyses were performed by sex and by age group (60–69 years and ≥ 70 years). In addition, we calculated the curve (AUC) of the receiver operating characteristic (ROC) and 95% CI through fitting logistic regression models and using the SPPB score as predictor. All data analyses were performed using SPSS, version 26.0 for Windows (SPSS, Inc., Chicago, IL, USA). Statistical significance level was set at 0.05.

## Results

As detailed in Fig. 1, our analysis included 9279, 6153, 4142, 4148, and 3583 eligible adults aged 60 years and older who completed follow-up assessments after 2, 3, 4, 5, and 7 years, respectively. The fall incidence among older adults per 100 person-years was 9.7% (95% CI: 9.3–10.1%) at the 2-year follow-up, 7.4% (95% CI: 7.0-7.7%) at the 3-year follow-up, 8.0% (95% CI: 7.6–8.4%) at the 4-year follow-up, 7.0% (95% CI: 6.7–7.4%) at the 5-year follow-up, and 6.2% (95% CI: 5.9–6.5%) at the 7-year follow-up. The corresponding fall-induced injury incidence per 100 person-years at the five follow-up time periods was 6.0% (95% CI: 5.6–6.3%), 4.6% (95% CI: 4.3–4.9%), 6.0% (95% CI: 5.6–6.3%), 5.4% (95% CI: 5.1–5.7%), and 5.4% (95% CI: 5.2–5.7%) (Appendix Table 2).

**Fig. 2:**
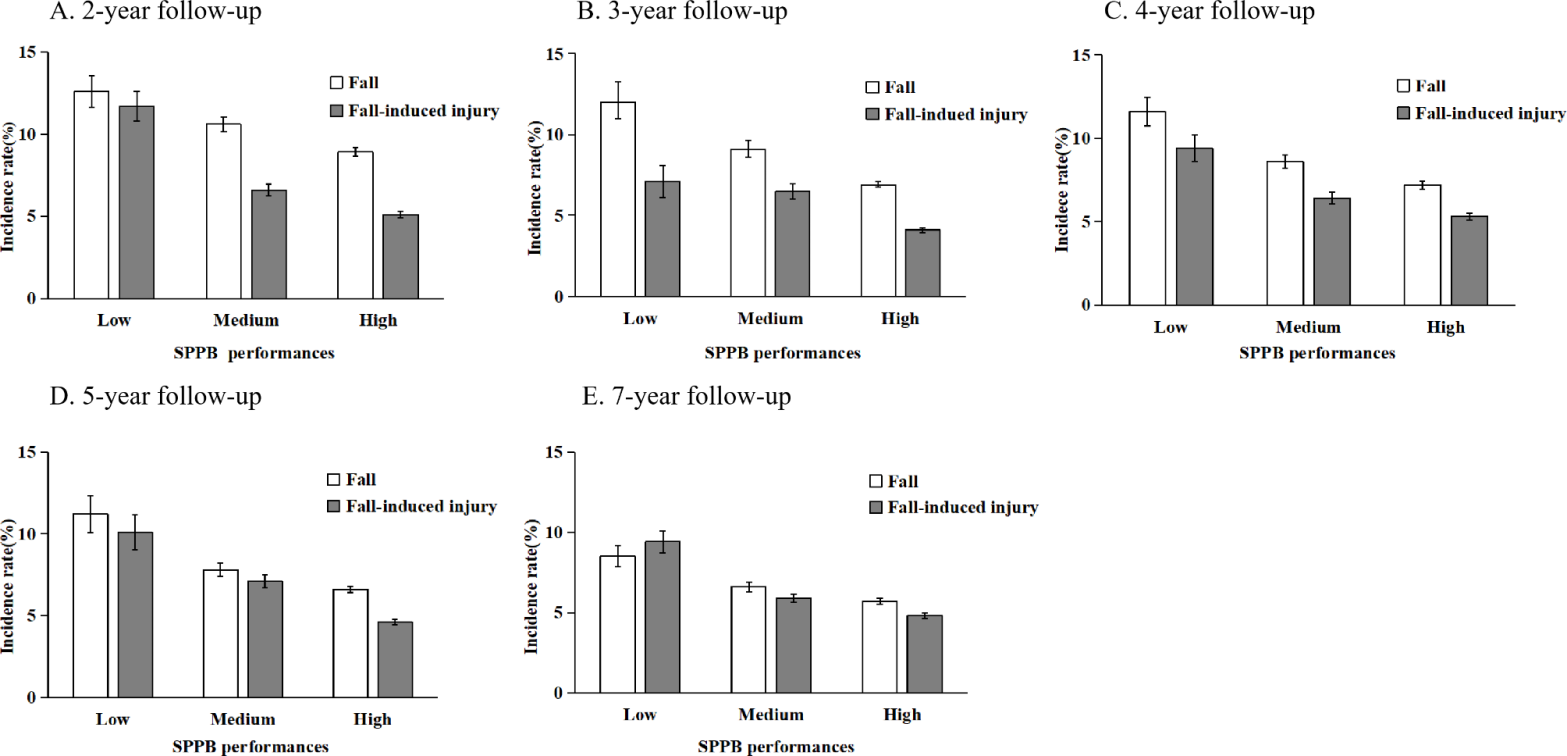
Incidence rates of fall and fall-induced injury among Chinese adults aged 60 years and older across different SPPB performances at five follow-up time periods. Notes: 1. Fall incidence rate was calculated as “(number of persons who experienced at least a fall / number of person-years x 100%)”, and fall-induced injury incidence rate was calculated as “(number of fall-induced injury / number of person-years x 100%)”. 2. All differences in fall incidence and in fall-induced incidence across the three SPPB performances were compared using Cochran-Armitage trend chi-square test and all were statistically significant, *p* < 0.05

As hypothesized, with covariates omitted from the model, both fall incidence and fall-induced injury incidence decreased significantly as SPPB performance increased; this was true at all five follow-up time periods, *P* < 0.05 (Fig. 2). Also as expected, older residents with high SSPB performance had a much lower incidence rate than those with low SPPB performance for both falls and fall-induced injuries.

After adjusting for sex, age group, ADL, history of fall in the past 2 years, depression, stroke, memory-related disease, sensory status, muscle weakness, and cognitive function, the SPPB performance was significantly associated with fall incidence only at the 4- and 5-year follow-up time periods, *P* < 0.05 (Table 1). After adjusting for the same covariates, SPPB performance was significantly associated with fall-induced injury at the 2, 5, and 7-year follow-up time periods (Table 2).

**Table 1:** Association of SPPB performances with fall incidence rates at different follow-up time periods

Follow-up time	SPPB performance	Number of person-years	Number	Incidence (%, 95% CI)	OR (95% CI)
2 years	High	12,506	1111	8.9% (8.4-9.4%)	1.00 (Ref.)
	Medium	4828	510	10.6% (9.7-11.4%)	1.01 (0.89–1.15)
	Low	1224	176	12.6% (10.8-14.3%)	1.19 (0.97–1.47)
3 years	High	15,189	1041	6.9% (6.5-7.3%)	1.00 (Ref.)
	Medium	2610	237	9.1% (8.0-10.2%)	1.04 (0.87–1.25)
	Low	660	79	12.0% (9.5-14.5%)	1.21 (0.88–1.67)
4 years	High	10,336	743	7.2% (6.7-7.7%)	1.00 (Ref.)
	Medium	4888	421	8.6% (7.8-9.4%)	1.10 (0.94–1.29)
	Low	1344	156	11.6% (9.9-13.3%)	1.42 (1.10–1.84) *
5 years	High	15,440	1014	6.6% (6.2-7.0%)	1.00 (Ref.)
	Medium	4515	352	7.8% (7.0-8.6%)	0.99 (0.84–1.17)
	Low	785	88	11.2% (9.0-13.4%)	1.54 (1.08–2.19) *
7 years	High	16,006	917	5.7% (5.4-6.1%)	1.00 (Ref.)
	Medium	7224	474	6.6% (6.0-7.1%)	1.02 (0.87–1.20)
	Low	1841	157	8.5% (7.3-9.8%)	1.31 (0.99–1.75)

Notes:

1. SPPB: Short Physical Performance Battery

2. Fall incidence rate was calculated as “(number of persons who experienced at least a fall / number of person-years◊100%)”

3. OR: Odds ratio, which was calculated after adjusting for sex, age group, ADL, history of fall in the past 2 years, depression, stroke, memory-related disease, sensory status, muscle weakness, and cognitive function

4. 95% CI: 95% confidence interval

5. ^*^: *P* < 0.05

**Table 2:** Association of SPPB performances with fall-induced injury incidence rates at different follow-up time periods

Follow-up time	SPPB performance	Number of person-years	Number	Incidence (%, 95% CI)	IRR (95%CI)
2 years	High	12506	644	5.1% (4.8-5.5%)	1.00 (Ref.)
	Medium	4828	319	6.6% (5.9-7.3%)	1.04 (0.89–1.21)
	Low	1224	143	11.7% (9.9-13.5%)	1.45 (1.16–1.81) *
3 years	High	15189	625	4.1% (3.8-4.4%)	1.00 (Ref.)
	Medium	2610	170	6.5% (5.6-7.5%)	1.12 (0.92–1.37)
	Low	660	47	7.1% (5.2-9.1%)	0.98 (0.69–1.41)
4 years	High	10336	552	5.3% (4.9-5.8%)	1.00 (Ref.)
	Medium	4888	311	6.4% (5.7-7.0%)	1.01 (0.86–1.19)
	Low	1344	126	9.4% (7.8-10.9%)	1.15 (0.90–1.48)
5 years	High	15440	714	4.6% (4.3-5.0%)	1.00 (Ref.)
	Medium	4515	322	7.1% (6.4-7.9%)	1.18 (1.00-1.38) *
	Low	785	79	10.1% (8.0-12.2%)	1.32 (0.97–1.80)
7 years	High	16006	765	4.8% (4.4-5.1%)	1.00 (Ref.)
	Medium	7224	427	5.9% (5.4-6.5%)	1.05 (0.90–1.21)
	Low	1841	173	9.4% (8.1-10.7%)	1.32 (1.05–1.66) *

Notes:

1. SPPB: Short Physical Performance Battery

2. Fall-induced injury incidence rate was calculated as “(number of fall-induced injury / number of person-years◊100%)”

3. 95% CI: 95% confidence interval

4. IRR: incidence rate ratio, which was calculated based on negative binomial regression models after adjusting for sex, age group, ADL, history of fall in the past 2 years, depression, stroke, memory-related disease, sensory status, muscle weakness, and cognitive function

5. ^*^: *P* < 0.05

The goodness-of-fit of predictive models using the SPPB performance as a single predictor was particularly low for both study outcome events across five follow-up time periods -- Cox-Snell R^2^: 0.003–0.011; Nagelkerke R^2^: 0.006–0.016; and accuracy: 0-10.1% for participants experiencing at least one fall or fall-induced injury, 94.8-100% for participants not experiencing a fall or fall-induced injury, and 58.2-91.6% for all participants combined. When using the SPPB performance and other variables as predictors together, the corresponding goodness-of-fit performance of predictive models was: Cox-Snell R^2^: 0.024–0.083; Nagelkerke R^2^: 0.055–0.114; and accuracy: 0-39.4% for participants experiencing at least one fall or fall-induced injury, 81.3-100% for participants not experiencing a fall or fall-induced injury, and 63.2-91.6% for all participants combined (Table 3). Subgroup analyses by sex and age group demonstrated highly similar predictive performance (Appendix Tables 3 and 4).

**Table 3:** Goodness-of-fit of predictive models for falls and fall-induced injuries based on multivariable logistic regression

Outcome event	Follow-up time	Model	Cox-Snell R^2^	Nagelkerke R^2^	Accuracy (%)		
					Group A	Group B	Combined
Fall	2 years	Model 1	0.005	0.008	0	100%	80.6%
		Model 2	0.049	0.078	2.0%	99.5%	80.6%
	3 years	Model 1	0.007	0.010	0	100%	77.9%
		Model 2	0.072	0.111	10.5%	97.4%	78.3%
	4 years	Model 1	0.011	0.016	0	100%	68.1%
		Model 2	0.061	0.085	18.0%	93.2%	69.2%
	5 years	Model 1	0.010	0.014	6.1%	97.4%	65.4%
		Model 2	0.083	0.114	28.1%	90.3%	68.5%
	7 years	Model 1	0.011	0.015	10.1%	94.8%	58.2%
		Model 2	0.072	0.096	39.4%	81.3%	63.2%
Fall-induced injury	2 years	Model 1	0.003	0.006	0	100%	91.6%
		Model 2	0.024	0.055	0	100%	91.6%
	3 years	Model 1	0.003	0.007	0	100%	90.6%
		Model 2	0.031	0.067	0	100%	90.6%
	4 years	Model 1	0.007	0.011	0	100%	84.9%
		Model 2	0.035	0.061	0	100%	84.9%
	5 years	Model 1	0.007	0.011	0	100%	83.8%
		Model 2	0.045	0.076	0.3%	99.9%	83.8%
	7 years	Model 1	0.010	0.015	0	100%	78.0%
		Model 2	0.052	0.079	3.8%	98.8%	78.0%

Notes:

1. Model 1 was fitted by including Short Physical Performance Battery (SPPB) performance as a single predictor

2. Model 2 was fitted by including SPPB performance, sex, age group, ADL, history of fall in the past 2 years, depression, stroke, memory-related disease, sensory status, muscle weakness, and cognitive function as predictors

3. The statistical test was significant for all predictive models at the 0.05 significance level

4. Group A denotes those experiencing a fall or a fall-induced injury at least once during the follow-up time period; group B denotes those not experiencing a fall or a fall-induced injury during the follow-up time periods; and the combined category denotes the combination of groups A and B

In addition, the area under the ROC curve for using the SPPB score as a single predictor ranged between 0.53 and 0.57 for predicting both fall and fall-induced injury across the five follow-up time periods for both sexes (Appendix Fig. 1 and Table 5). While using the SPPB score and other predictors simultaneously, the area under the ROC curve for predicting fall and fall-induced injury increased to a range of 0.61–0.67. Age-specific analyses for the 60–69 years and 70 years and older groups displayed highly similar results (Appendix Tables 6 and 7).

## Discussion

### Primary findings

Using nationally representative cohort data, we evaluated longitudinally the validity of the SPPB to predict subsequent falls and fall-induced injury among Chinese dwellers aged ≥ 60 years at five follow-up time periods. Four key findings emerged: (i) after adjusting for the included covariates, SPPB performance was significantly associated with fall and fall-induced injury at some but not all of the five follow-up time periods; (ii) whether using SPPB performance as a single ordinal-variable predictor or using SPPB performance and other risk factors as predictors together, the goodness-of-fit of models predicting fall and fall-induced injury was low; (iii) when using the SPPB score as a continuous-variable predictor, the AUCs of univariable and multivariable predictive models were all less than 0.70; and (iv) the goodness-of-fit of models to predict fall and fall-induced injury were similar across sex- and age-specific analyses.

### Interpretation of findings

Surprisingly, the multivariable analyses did not detect strong positive associations between SPPB and the follow-up study outcome measures, contradicting the findings of our univariable analyses as well as previous reports that showed significant associations between SPPB and the number of falls [11] and the occurrence of recurrent falls [10]. Our results do concord with findings from Ward et al. [12] and suggest SPPB performance may be less meaningful in predicting the occurrence of future falls and fall-induced injuries when used alone compared to when it is used along with relevant demographic variables and self-reported health conditions as predictors.

Quantitative evidence supports the above inference. Consistent with two prior studies [13, 14], goodness-of-fit of the predictive models in our research did not reach the common criteria for predictive models [30]. The low goodness-of-fit of predictive models may be due to several possible reasons. First, the CHARLS study used self-reported indicators to measure individual health conditions and reporting bias may have impacted results. Second, the CHARLS study did not collect biological data and thus our predictive models omitted potentially-relevant genetic factors [31]. Third, ceiling effects that can emerge with the typical scoring system for the SPPB may have impacted on its predictive performance. Following patterns from the Established Populations for Epidemiological Studies in the Elderly (EPESE) [32], the SPPB score is traditionally divided into three levels of performances (low, medium, high), with a large portion of community-dwelling participants falling into the high SPPB performance category. Fukui et al. [33] reported similar ceiling effects with the SPPB scoring system and interpreted them as one possible reason for insufficient performance in SPPB scores predicting future falls and fall-induced injuries.

Subgroup analyses by sex and age group in our study generated nearly identical predictive performance to what we found in our overall sample. This finding differed from a previous cross-sectional study of 7474 home dwellers aged 40 years and older in Norway that found SPPB performance declined with age and varied between females and males [34]. The inconsistency may be caused by differences in the study samples, the incidence of primary outcomes, and the covariates included in predictive models. Our study considered 9 demographic variables and self-reported health conditions as covariates across two age groupings while the Bergland and Strand study included only education level and Body Mass Index (BMI) as covariates but considered five age groupings [34].

### Implications

Our findings have two important implications. First, they affirm that neither sole use of SPPB scores nor joint use of SPPB scores with demographic variables and self-reported health conditions achieves acceptable criteria for predictive models or as diagnostic tools (e.g., AUC ≥ 0.70 and accuracy ≥ 70%). We therefore do not recommend use of the SPPB tool alone to predict future falls and fall-induced injuries among older community-dwelling Chinese adults. Nevertheless, the SPPB is a useful and convenient tool that can be used as a rough screen for the risk of falls and fall-induced injury among elderly individuals, particularly to detect physical function weaknesses like speed and balance. Second, considering the wide current use of SPPB, further research is recommended to explore possible reasons for insufficient predictive performance of the SPPB and to develop solutions to improve its predictive performance, such as integrating new biomarkers as co-predictors, revising the SPPB components or scoring scheme, or adopting more precise measures of physical function.

### Study limitations

Our study has several limitations. First, the incidence measures of falls and fall-induced injuries were based on the participant’s self-reports and may be underestimated because of recall bias [35]. Second, missing values might have some impact on our results because some might not be missing at random, as assumed. Third, potentially important covariate factors such as medications and genetic factors were excluded from our analysis because they were not assessed in the CHARLS study.

## Conclusion

In summary, the SPPB was found to have insufficient capacity to predict future falls or fall-induced injuries among older Chinese adults. Consequently, we do not recommend using the SPPB alone to predict of future fall and fall-induced injury among old community-dwelling Chinese adults. We suggest further research to explore the reasons for insufficient prediction performance and to develop solutions to increase the predictive value of the SPPB.

### Electronic supplementary material

Below is the link to the electronic supplementary material.

Supplementary Material 1

## Data Availability

The CHARLS datasets, which analyzed during the current study, are publicly available at the National School of Development, Peking University (http://charls.pku.edu.cn/) and can be obtained after submitting a data use agreement to the CHARLS team.

## Abbreviations

SPPB: Short Physical Performance Battery
CHARLS: China Health and Retirement Longitudinal Study
AUC: area under the receiver operating curve
ADL: Activities of daily living
CESD-10: 10-item version of the Center for Epidemiological Scale
BMI: body mass index
HRS: Health and Retirement Study
OR: odds ratio
IRR: incidence rate ratio
ROC: receiver operating characteristic
