# Supplementary material for: Value of the short physical performance battery (SPPB) in predicting fall and fall-induced injury among old Chinese adults
Source: BMC Geriatr. 2023 Sep 18;23:574. doi: 10.1186/s12877-023-04290-6 (PMC10507826; doi:10.1186/s12877-023-04290-6)
Supplement: Supplementary file 1 — Supplementary Material 1 [file 12877_2023_4290_MOESM1_ESM.docx]

**Appendix Tables and Figures**

**Table 1.** Baseline characteristics of the CHARLS participants at five follow-up time periods

| Variable | 2 years (n=9279) | 3 years (n=6153) | 4 years (n=4142) | 5 years (n=4148) | 7 years (n=3583) |
| --- | --- | --- | --- | --- | --- |
| Age, n (%) |  |  |  |  |  |
| 60-69 years | 6300 (67.8) | 2364 (67.1) | 2879 (69.5) | 2897 (69.8) | 2585 (72.1) |
| ≥70 years | 2979 (32.1) | 2022 (32.8) | 1263 (30.4) | 1251 (30.1) | 998 (27.8) |
| Median (IQR) | 66 (9) | 66 (8) | 66 (9) | 66 (9) | 65 (8) |
| Gender, n (%) |  |  |  |  |  |
| Male | 4770 (51.4) | 3041 (49.4) | 2101 (50.7) | 2094 (50.5) | 1767 (49.3) |
| Female | 4509 (48.6) | 3112 (50.6) | 2041 (49.3) | 2054 (49.5) | 1816 (50.7) |
| ADL, n (%) |  |  |  |  |  |
| Not impaired | 7570 (81.6) | 4779 (77.7) | 3313 (80.0) | 3344 (80.6) | 2875 (80.2) |
| Impaired | 1709 (18.4) | 1374 (22.3) | 829 (20.0) | 804 (19.4) | 708 (19.8) |
| Depression, n (%) |  |  |  |  |  |
| No depressive symptoms | 5988 (64.5) | 4095 (66.6) | 2507 (60.5) | 2811 (67.8) | 2190 (61.1) |
| Depressive symptoms | 3291 (35.5) | 2058 (33.4) | 1635 (39.5) | 1337 (32.2) | 1393 (38.9) |
| Fall in the past 2 years, n (%) |  |  |  |  |  |
| No | 7570 (81.6) | 4968 (80.7) | 3342 (80.7) | 3407 (82.1) | 2882 (80.4) |
| Yes | 1709 (18.4) | 1185 (19.3) | 800 (19.3) | 741 (17.9) | 701 (19.6) |
| Stroke, n (%) |  |  |  |  |  |
| No | 9053 (97.6) | 6028 (98.0) | 4039 (97.5) | 4061(97.9) | 3495 (97.5) |
| Yes | 226 (2.4) | 125 (2.0) | 103 (2.5) | 87 (2.1) | 88 (2.5) |
| Memory-related diseases, n (%) |  |  |  |  |  |
| No | 9127 (98.4) | 6041 (98.2) | 4065 (98.1) | 4099 (98.8) | 3521 (98.3) |
| Yes | 152 (1.6) | 112 (1.8) | 77 (1.9) | 49 (1.2) | 62 (1.7) |
| Sensory status, n (%) |  |  |  |  |  |
| No sensory loss | 1130 (12.2) | 613 (10.0) | 532 (12.8) | 491 (11.8) | 466 (13.0) |
| Hearing loss | 634 (6.8) | 349 (5.7) | 262 (6.3) | 294 (7.1) | 224 (6.3) |
| Vision loss | 2135 (23.0) | 1173 (19.1) | 991 (23.9) | 924 (22.3) | 855 (23.9) |
| Both hearing and vision loss | 5380 (58) | 4018 (65.3) | 2357 (56.9) | 2439 (58.8) | 2038 (56.9) |
| Muscle weakness, n (%) |  |  |  |  |  |
| No | 7815 (84.2) | 5005 (81.3) | 3500 (84.5) | 3561 (85.8) | 3061 (85.4) |
| Yes | 1464 (15.8) | 1148 (18.7) | 642 (15.5) | 587 (14.2) | 522 (14.6) |
| Cognitive function, n (%) |  |  |  |  |  |
| <*P_25_* | 2458 (26.5) | 1570 (25.5) | 1120 (27.0) | 1037 (25.0) | 939 (26.2) |
| *P_25_*-*P_50_* | 2729 (29.4) | 1695 (27.5) | 1174 (28.3) | 1285 (31.0) | 1034 (28.9) |
| *P_50_*-*P_75_* | 2273 (24.5) | 1526 (24.8) | 987 (23.8) | 803 (19.4) | 851 (23.8) |
| ≥*P_75_* | 1819 (19.6) | 1362 (22.1) | 861 (20.8) | 1023 (24.7) | 759 (21.2) |
| Mean (SD) | 12.3 (5.66) | 11.7 (5.68) | 12.4 (5.82) | 12.4 (5.50) | 12.4 (5.79) |
| SPPB, n (%) |  |  |  |  |  |
| Low | 612 (6.6) | 220 (3.6) | 336 (8.1) | 157 (3.8) | 263 (7.3) |
| Medium | 2414 (26.0) | 870 (14.1) | 1222 (29.5) | 903 (21.8) | 1032 (28.8) |
| High | 6253 (67.4) | 5063 (82.3) | 2584 (62.4) | 3088 (74.4) | 2288 (63.9) |
| Mean (SD) | 10.0 (2.03) | 10.7 (1.74) | 9.7 (2.11) | 10.3 (1.80) | 9.8 (2.04) |
| Fall, n (%) |  |  |  |  |  |
| No | 7482 (80.6) | 4796 (77.9) | 2822 (68.1) | 2694 (64.9) | 2035 (56.8) |
| Yes | 1797 (19.4) | 1357 (22.1) | 1320 (31.9) | 1454 (35.1) | 1548 (43.2) |
| Number of fall-induced injuries, n (%) |  |  |  |  |  |
| 0 | 8501 (91.6) | 5575 (90.6) | 3518 (84.9) | 3476 (83.8) | 2796 (78.0) |
| ≥1 | 778 (8.4) | 578 (9.4) | 624 (15.1) | 672 (16.2) | 787 (22.0) |

Notes: CHARLS: China Health and Retirement Longitudinal Study; ADL: activities of daily living; SPPB: Short Physical Performance Battery.

**Table 2.** Fall and fall-induced incidence rates at five CHARLS follow-up time periods

| Outcome event | Follow-up time | Number of person-years | Number | Incidence (%, 95% CI) |
| --- | --- | --- | --- | --- |
| Fall | 2 years | 18558 | 1797 | 9.7% (9.3%-10.1%) |
|  | 3 years | 18459 | 1357 | 7.4% (7.0%-7.7%) |
|  | 4 years | 16568 | 1320 | 8.0% (7.6%-8.4%) |
|  | 5 years | 20740 | 1454 | 7.0% (6.7%-7.4%) |
|  | 7 years | 25081 | 1548 | 6.2% (5.9%-6.5%) |
| Fall-induced injury | 2 years | 18558 | 1106 | 6.0% (5.6%-6.3%) |
|  | 3 years | 18459 | 842 | 4.6% (4.3%-4.9%) |
|  | 4 years | 16568 | 989 | 6.0% (5.6%-6.3%) |
|  | 5 years | 20740 | 1115 | 5.4% (5.1%-5.7%) |
|  | 7 years | 25081 | 1365 | 5.4% (5.2%-5.7%) |

Notes:

1. CHARLS: China Health and Retirement Longitudinal Study.

2. Fall incidence rate was calculated as “(number of persons who experienced at least a fall / number of person-years×100%)”.

3. Fall-induced injury incidence rate was calculation as “(number of fall-induced injury / number of person-years×100%)”.

**Table 3.** Goodness-fit-of predictive models based on multivariable logistic regression by outcome and sex

| Outcome event | Follow-up time | Sex | Model | Cox-Snell R^2^ | Nagelkerke R^2^ | Accuracy (%) | | |
| --- | --- | --- | --- | --- | --- | --- | --- | --- |
|  |  |  |  |  |  | Group A | Group B | Combined |
| Fall | 2 years | Male | Model 1 | 0.004 | 0.006 | 0 | 100% | 84.1% |
|  |  |  | Model 2 | 0.042 | 0.073 | 0.3% | 99.9% | 84.1% |
|  |  | Female | Model 1 | 0.003 | 0.005 | 0 | 100% | 76.9% |
|  |  |  | Model 2 | 0.042 | 0.063 | 1.5% | 99.5% | 77.0% |
|  | 3 years | Male | Model 1 | 0.008 | 0.012 | 0 | 100% | 82.3% |
|  |  |  | Model 2 | 0.048 | 0.079 | 2.8% | 99.5% | 82.4% |
|  |  | Female | Model 1 | 0.003 | 0.005 | 0 | 100% | 73.7% |
|  |  |  | Model 2 | 0.078 | 0.114 | 15.9% | 95.7% | 74.7% |
|  | 4 years | Male | Model 1 | 0.007 | 0.010 | 0 | 100% | 72.9% |
|  |  |  | Model 2 | 0.058 | 0.084 | 11.6% | 96.7% | 73.7% |
|  |  | Female | Model 1 | 0.009 | 0.013 | 0 | 100% | 63.2% |
|  |  |  | Model 2 | 0.051 | 0.069 | 23.4% | 90.0% | 65.5% |
|  | 5 years | Male | Model 1 | 0.009 | 0.012 | 4.4% | 98.5% | 71.3% |
|  |  |  | Model 2 | 0.060 | 0.086 | 14.7% | 95.4% | 72.0% |
|  |  | Female | Model 1 | 0.006 | 0.008 | 7.2% | 96.1% | 59.4% |
|  |  |  | Model 2 | 0.076 | 0.102 | 38.7% | 85.0% | 65.9% |
|  | 7 years | Male | Model 1 | 0.009 | 0.013 | 6.0% | 97.2% | 63.7% |
|  |  |  | Model 2 | 0.053 | 0.073 | 25.3% | 89.4% | 65.9% |
|  |  | Female | Model 1 | 0.007 | 0.009 | 13.1% | 91.8% | 52.9% |
|  |  |  | Model 2 | 0.057 | 0.076 | 51.1% | 67.9% | 59.6% |
| Fall-induced injury | 2 years | Male | Model 1 | 0.002 | 0.006 | 0 | 100% | 93.7% |
|  |  |  | Model 2 | 0.024 | 0.065 | 0 | 100% | 93.7% |
|  |  | Female | Model 1 | 0.001 | 0.003 | 0 | 100% | 89.4% |
|  |  |  | Model 2 | 0.015 | 0.031 | 0 | 100% | 89.4% |
|  | 3 years | Male | Model 1 | 0.001 | 0.003 | 0 | 100% | 93.2% |
|  |  |  | Model 2 | 0.015 | 0.037 | 0 | 100% | 93.2% |
|  |  | Female | Model 1 | 0.003 | 0.005 | 0 | 100% | 88.1% |
|  |  |  | Model 2 | 0.036 | 0.069 | 0 | 100% | 88.1% |
|  | 4 years | Male | Model 1 | 0.003 | 0.007 | 0 | 100% | 88.4% |
|  |  |  | Model 2 | 0.031 | 0.060 | 0 | 100% | 88.4% |
|  |  | Female | Model 1 | 0.005 | 0.008 | 0 | 100% | 81.3% |
|  |  |  | Model 2 | 0.026 | 0.042 | 0 | 100% | 81.3% |
|  | 5 years | Male | Model 1 | 0.002 | 0.003 | 0 | 100% | 87.9% |
|  |  |  | Model 2 | 0.038 | 0.073 | 0 | 100% | 87.9% |
|  |  | Female | Model 1 | 0.007 | 0.011 | 0 | 100% | 79.6% |
|  |  |  | Model 2 | 0.034 | 0.054 | 0 | 99.9% | 79.6% |
|  | 7 years | Male | Model 1 | 0.003 | 0.006 | 0 | 100% | 83.4% |
|  |  |  | Model 2 | 0.032 | 0.054 | 0 | 99.9% | 83.4% |
|  |  | Female | Model 1 | 0.009 | 0.013 | 0 | 100% | 72.8% |
|  |  |  | Model 2 | 0.041 | 0.060 | 3.0% | 98.6% | 72.6% |

Notes:

1. Model 1 was fitted by including Short Physical Performance Battery (SPPB) performance as a single predictor.

2. Model 2 was fitted by including SPPB performance, age group, ADL, history of fall in the past 2 years, depression, stroke, memory-related disease, sensory status, muscle weakness, and cognitive function as predictors.

3. The statistical test was significant for all predictive models at the 0.05 significance level.

4. Group A denotes those experiencing a fall or a fall-induced injury at least once during the follow-up time period; group B denotes those not experiencing a fall or a fall-induced injury during the follow-up time periods; and the combined category denotes the combination of groups A and B.

**Table 4.** Goodness-fit-of predictive models based on multivariable logistic regression by outcome and age group

| Outcome event | Follow-up time | Age group | Model | Cox-Snell R^2^ | Nagelkerke R^2^ | Accuracy (%) | | |
| --- | --- | --- | --- | --- | --- | --- | --- | --- |
|  |  |  |  |  |  | Group A | Group B | Combined |
| Fall | 2 years | 60-69 years | Model 1 | 0.006 | 0.009 | 0 | 100% | 81.6% |
|  |  |  | Model 2 | 0.045 | 0.074 | 1.5% | 99.6% | 81.5% |
|  |  | ≥70 years | Model 1 | 0.003 | 0.004 | 0 | 100% | 78.6% |
|  |  |  | Model 2 | 0.042 | 0.065 | 0.6% | 99.7% | 78.5% |
|  | 3 years | 60-69 years | Model 1 | 0.007 | 0.011 | 0 | 100% | 79.8% |
|  |  |  | Model 2 | 0.074 | 0.117 | 10.8% | 97.8% | 80.2% |
|  |  | ≥70 years | Model 1 | 0.004 | 0.005 | 0 | 100% | 74.2% |
|  |  |  | Model 2 | 0.055 | 0.080 | 10.9% | 96.8% | 74.6% |
|  | 4 years | 60-69 years | Model 1 | 0.012 | 0.017 | 0 | 100% | 69.3% |
|  |  |  | Model 2 | 0.058 | 0.082 | 15.1% | 94.8% | 70.3% |
|  |  | ≥70 years | Model 1 | 0.007 | 0.010 | 0 | 100% | 65.4% |
|  |  |  | Model 2 | 0.057 | 0.078 | 23.3% | 89.8% | 66.8% |
|  | 5 years | 60-69 years | Model 1 | 0.007 | 0.009 | 3.6% | 98.5% | 67.0% |
|  |  |  | Model 2 | 0.084 | 0.117 | 26.2% | 92.0% | 70.2% |
|  |  | ≥70 years | Model 1 | 0.012 | 0.017 | 10.8% | 94.9% | 61.7% |
|  |  |  | Model 2 | 0.058 | 0.079 | 28.4% | 86.7% | 63.7% |
|  | 7 years | 60-69 years | Model 1 | 0.010 | 0.013 | 7.4% | 96.4% | 60.4% |
|  |  |  | Model 2 | 0.055 | 0.074 | 30.6% | 85.1% | 63.0% |
|  |  | ≥70 years | Model 1 | 0.007 | 0.010 | 52.1% | 53.1% | 52.6% |
|  |  |  | Model 2 | 0.064 | 0.086 | 50.1% | 69.2% | 59.6% |
| Fall-induced injury | 2 years | 60-69 years | Model 1 | 0.003 | 0.007 | 0 | 100% | 92.0% |
|  |  |  | Model 2 | 0.022 | 0.052 | 0 | 100% | 92.0% |
|  |  | ≥70 years | Model 1 | 0.002 | 0.004 | 0 | 100% | 90.8% |
|  |  |  | Model 2 | 0.019 | 0.042 | 0 | 100% | 90.8% |
|  | 3 years | 60-69 years | Model 1 | 0.004 | 0.008 | 0 | 100% | 91.6% |
|  |  |  | Model 2 | 0.028 | 0.064 | 0 | 100% | 91.6% |
|  |  | ≥70 years | Model 1 | 0.002 | 0.003 | 0 | 100% | 88.6% |
|  |  |  | Model 2 | 0.029 | 0.058 | 0 | 100% | 88.6% |
|  | 4 years | 60-69 years | Model 1 | 0.005 | 0.010 | 0 | 100% | 85.7% |
|  |  |  | Model 2 | 0.035 | 0.063 | 0 | 100% | 85.7% |
|  |  | ≥70 years | Model 1 | 0.007 | 0.012 | 0 | 100% | 83.1% |
|  |  |  | Model 2 | 0.026 | 0.044 | 0 | 100% | 83.1% |
|  | 5 years | 60-69 years | Model 1 | 0.005 | 0.010 | 0 | 100% | 84.9% |
|  |  |  | Model 2 | 0.043 | 0.076 | 0 | 99.8% | 84.8% |
|  |  | ≥70 years | Model 1 | 0.006 | 0.010 | 0 | 100% | 81.1% |
|  |  |  | Model 2 | 0.035 | 0.057 | 0.4% | 99.8% | 81.4% |
|  | 7 years | 60-69 years | Model 1 | 0.006 | 0.009 | 0 | 100% | 80.1% |
|  |  |  | Model 2 | 0.045 | 0.071 | 1.8% | 99.5% | 80.1% |
|  |  | ≥70 years | Model 1 | 0.013 | 0.019 | 0 | 100% | 72.6% |
|  |  |  | Model 2 | 0.043 | 0.062 | 4.8% | 97.5% | 72.1% |

Notes:

1. Model 1 was fitted by including Short Physical Performance Battery (SPPB) performance as a single predictor.

2. Model 2 was fitted by including SPPB performance, sex, ADL, history of fall in the past 2 years, depression, stroke, memory-related disease, sensory status, muscle weakness, and cognitive function as predictors.

3. The statistical test was significant for all predictive models at the 0.05 significance level.

4. Group A denotes those experiencing a fall or a fall-induced injury at least once during the follow-up time period; group B denotes those not experiencing a fall or a fall-induced injury during the follow-up time periods; and the combined category denotes the combination of groups A and B.

**
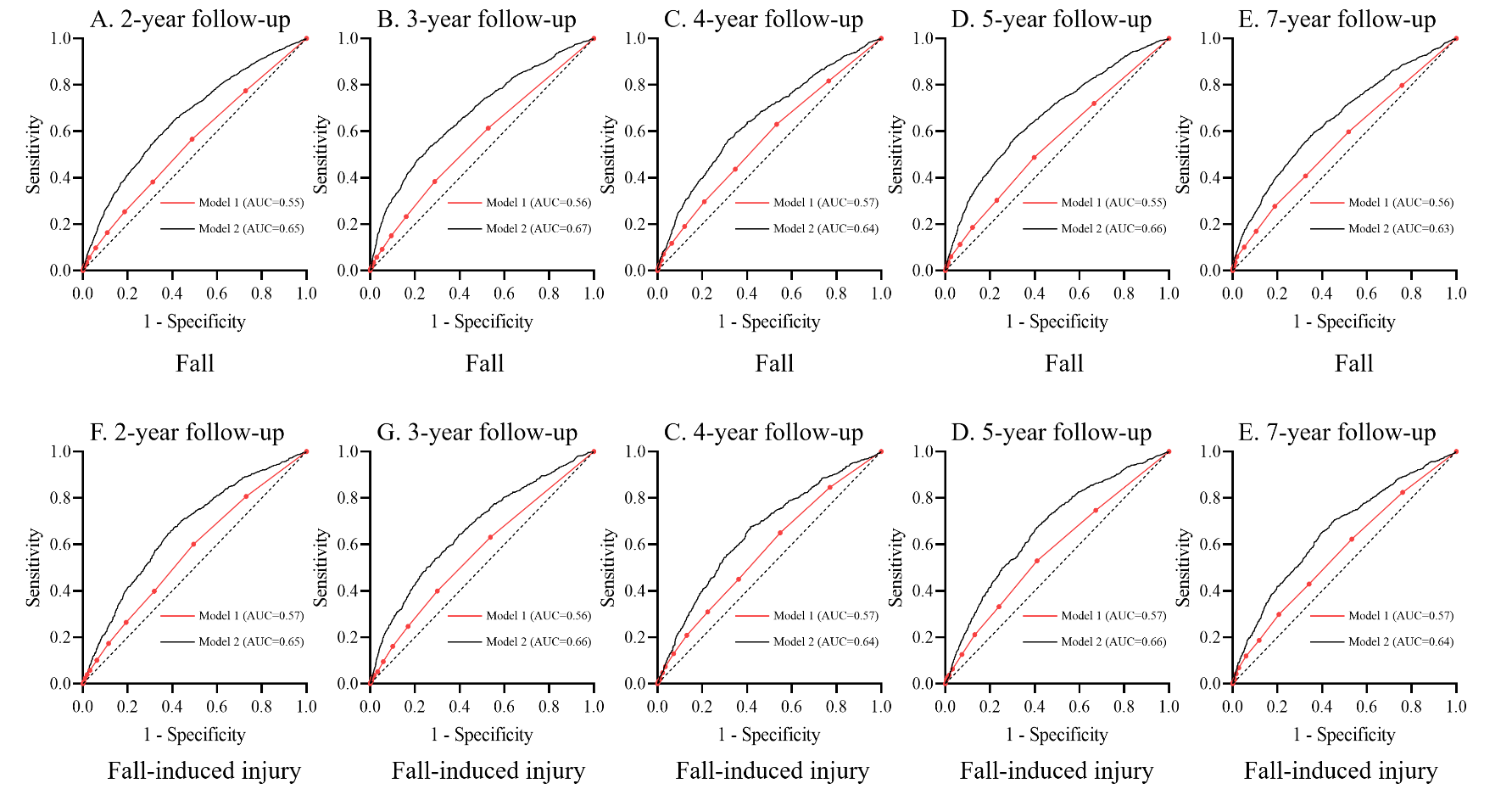
**

**Figure 1.** Receiver operating characteristic curves of using SPPB to predict falls and fall-induced injuries at different follow-up time periods.

Notes:

1. Model 1 was fitted by including Short Physical Performance Battery (SPPB) score as a single predictor.

2. Model 2 was fitted by including SPPB score, sex, age group, ADL, history of fall in the past 2 years, depression, stroke, memory-related disease, sensory status, muscle weakness, and cognitive function as predictors.

3. AUC: area under the curve.

**Table 5.** Area under the ROC curve and 95% confidence interval for predictive models based on multivariable logistic regression by outcome and sex

| Outcome event | Sex | Model | Follow-up time | | | | |
| --- | --- | --- | --- | --- | --- | --- | --- |
|  |  |  | 2 years | 3 years | 4 years | 5 years | 7 years |
| Fall | Both sexes | Model 1 | 0.55 (0.54-0.57) | 0.56 (0.54-0.58) | 0.57 (0.55-0.58) | 0.55 (0.54-0.57) | 0.56 (0.54-0.58) |
|  |  | Model 2 | 0.65 (0.63-0.66) | 0.67 (0.65-0.69) | 0.64 (0.62-0.66) | 0.66 (0.64-0.68) | 0.63 (0.61-0.65) |
|  | Male | Model 1 | 0.54 (0.52-0.56) | 0.55 (0.53-0.58) | 0.55 (0.52-0.58) | 0.55 (0.52-0.58) | 0.55 (0.52-0.57) |
|  |  | Model 2 | 0.66 (0.62-0.69) | 0.62 (0.58-0.66) | 0.63 (0.59-0.67) | 0.65 (0.61-0.69) | 0.61 (0.57-0.65) |
|  | Female | Model 1 | 0.54 (0.52-0.56) | 0.54 (0.52-0.57) | 0.56 (0.53-0.58) | 0.54 (0.51-0.56) | 0.54 (0.52-0.57) |
|  |  | Model 2 | 0.63 (0.61-0.65) | 0.67 (0.65-0.70) | 0.63 (0.61-0.66) | 0.66 (0.63-0.68) | 0.63 (0.61-0.66) |
| Fall-induced injury | Both sexes | Model 1 | 0.57 (0.55-0.59) | 0.56 (0.54-0.59) | 0.57 (0.55-0.60) | 0.57 (0.55-0.59) | 0.57 (0.54-0.59) |
|  |  | Model 2 | 0.65 (0.63-0.67) | 0.66 (0.63-0.68) | 0.64 (0.62-0.67) | 0.66 (0.63-0.68) | 0.64 (0.62-0.66) |
|  | Male | Model 1 | 0.56 (0.52-0.59) | 0.55 (0.51-0.59) | 0.56 (0.52-0.59) | 0.54 (0.51-0.58) | 0.53 (0.50-0.57) |
|  |  | Model 2 | 0.67 (0.64-0.70) | 0.64 (0.60-0.68) | 0.65 (0.61-0.69) | 0.66 (0.62-0.70) | 0.64 (0.60-0.67) |
|  | Female | Model 1 | 0.55 (0.52-0.58) | 0.55 (0.52-0.58) | 0.56 (0.53-0.59) | 0.57 (0.54-0.60) | 0.56 (0.53-0.59) |
|  |  | Model 2 | 0.62 (0.59-0.64) | 0.66 (0.63-0.69) | 0.63 (0.60-0.66) | 0.63 (0.60-0.66) | 0.63 (0.60-0.66) |

Notes:

1. Model 1 was fitted by including Short Physical Performance Battery (SPPB) score as a single predictor.

2. Model 2 was fitted by including SPPB score, age group, ADL, history of fall in the past 2 years, depression, stroke, memory-related disease, sensory status, muscle weakness, and cognitive function as predictors.

3. The statistical test was significant for all predictive models at the 0.05 significance level.

**Table 6.** Area under the ROC curve and 95% confidence interval based on multivariable logistic regression in older Chinese adults aged 60-69 years

| Outcome event | Sex | Model | Follow-up time | | | | |
| --- | --- | --- | --- | --- | --- | --- | --- |
|  |  |  | 2 years | 3 years | 4 years | 5 years | 7 years |
| Fall | Both sexes | Model 1 | 0.55(0.53-0.56) | 0.56(0.54-0.58) | 0.56(0.54-0.59) | 0.55(0.52-0.57) | 0.54(0.52-0.57) |
|  |  | Model 2 | 0.65(0.62-0.68) | 0.65(0.62-0.68) | 0.65(0.62-0.68) | 0.66(0.63-0.68) | 0.63(0.60-0.66) |
|  | Male | Model 1 | 0.53(0.50-0.56) | 0.57(0.53-0.60) | 0.54(0.51-0.58) | 0.54(0.51-0.57) | 0.52(0.49-0.55) |
|  |  | Model 2 | 0.65(0.62-0.68) | 0.66(0.63-0.70) | 0.63(0.60-0.67) | 0.66(0.62-0.69) | 0.61(0.57-0.64) |
|  | Female | Model 1 | 0.54(0.51-0.56) | 0.54(0.51-0.57) | 0.56(0.53-0.59) | 0.52(0.49-0.55) | 0.54(0.51-0.57) |
|  |  | Model 2 | 0.65(0.62-0.67) | 0.69(0.66-0.72) | 0.64(0.61-0.67) | 0.66(0.64-0.69) | 0.64(0.61-0.67) |
| Fall-induced injury | Both sexes | Model 1 | 0.56(0.54-0.59) | 0.56(0.53-0.59) | 0.57(0.54-0.60) | 0.56(0.53-0.59) | 0.55(0.52-0.58) |
|  |  | Model 2 | 0.66(0.63-0.68) | 0.66(0.63-0.69) | 0.65(0.62-0.68) | 0.66(0.64-0.69) | 0.64(0.61-0.67) |
|  | Male | Model 1 | 0.54(0.50-0.58) | 0.56(0.51-0.61) | 0.55(0.50-0.60) | 0.53(0.48-0.57) | 0.51(0.46-0.55) |
|  |  | Model 2 | 0.67(0.63-0.71) | 0.62(0.57-0.67) | 0.67(0.62-0.71) | 0.67(0.62-0.71) | 0.65(0.61-0.70) |
|  | Female | Model 1 | 0.55(0.52-0.59) | 0.54(0.50-0.58) | 0.55(0.52-0.59) | 0.55(0.51-0.59) | 0.55(0.52-0.59) |
|  |  | Model 2 | 0.64(0.61-0.67) | 0.68(0.65-0.72) | 0.64(0.60-0.67) | 0.64(0.61-0.68) | 0.65(0.61-0.68) |

Notes:

1. Model 1 was fitted by including Short Physical Performance Battery (SPPB) score as a single predictor.

2. Model 2 was fitted by including SPPB score, ADL, history of fall in the past 2 years, depression, stroke, memory-related disease, sensory status, muscle weakness, and cognitive function as predictors.

3. The statistical test was significant for all predictive models at the significance level of 0.05.

**Table 7.** Area under the ROC curve and 95% confidence interval based on multivariable logistic regression in older Chinese adults aged 70 years and older

| Outcome event | Sex | Model | Follow-up time | | | | |
| --- | --- | --- | --- | --- | --- | --- | --- |
|  |  |  | 2 years | 3 years | 4 years | 5 years | 7 years |
| Fall | Both sexes | Model 1 | 0.55(0.52-0.57) | 0.53(0.51-0.56) | 0.56(0.53-0.59) | 0.56(0.53-0.59) | 0.56(0.52-0.59) |
|  |  | Model 2 | 0.63(0.60-0.67) | 0.64(0.61-0.68) | 0.62(0.58-0.66) | 0.63(0.59-0.67) | 0.61(0.57-0.65) |
|  | Male | Model 1 | 0.54(0.51-0.58) | 0.51(0.47-0.55) | 0.57(0.52-0.61) | 0.54(0.50-0.59) | 0.57(0.52-0.62) |
|  |  | Model 2 | 0.66(0.63-0.70) | 0.63(0.59-0.67) | 0.67(0.63-0.72) | 0.63(0.58-0.67) | 0.65(0.60-0.70) |
|  | Female | Model 1 | 0.52(0.49-0.56) | 0.52(0.48-0.56) | 0.52(0.48-0.57) | 0.55(0.50-0.59) | 0.52(0.47-0.57) |
|  |  | Model 2 | 0.62(0.58-0.65) | 0.66(0.62-0.69) | 0.64(0.59-0.68) | 0.67(0.63-0.71) | 0.64(0.59-0.69) |
| Fall-induced injury | Both sexes | Model 1 | 0.56(0.52-0.59) | 0.54(0.50-0.58) | 0.57(0.53-0.61) | 0.57(0.54-0.61) | 0.57(0.53-0.61) |
|  |  | Model 2 | 0.63(0.60-0.67) | 0.65(0.62-0.69) | 0.63(0.59-0.68) | 0.64(0.60-0.68) | 0.64(0.61-0.68) |
|  | Male | Model 1 | 0.56(0.50-0.61) | 0.51(0.44-0.57) | 0.56(0.49-0.62) | 0.54(0.48-0.60) | 0.55(0.49-0.62) |
|  |  | Model 2 | 0.69(0.64-0.74) | 0.67(0.61-0.72) | 0.64(0.57-0.71) | 0.66(0.60-0.72) | 0.63(0.58-0.69) |
|  | Female | Model 1 | 0.53(0.49-0.58) | 0.53(0.48-0.58) | 0.55(0.50-0.61) | 0.57(0.52-0.63) | 0.55(0.50-0.61) |
|  |  | Model 2 | 0.61(0.56-0.66) | 0.64(0.59-0.69) | 0.64(0.58-0.69) | 0.62(0.56-0.67) | 0.63(0.58-0.68) |

Notes:

1. Model 1 was fitted by including Short Physical Performance Battery (SPPB) score as a single predictor.

2. Model 2 was fitted by including SPPB score, ADL, history of fall in the past 2 years, depression, stroke, memory-related disease, sensory status, muscle weakness, and cognitive function as predictors.

3. The statistical test was significant for all predictive models at the significance level of 0.05.
